# Impact of Comorbidities and Previous Surgery on Mid-Term Results of Revision Total Knee Arthroplasty for Periprosthetic Joint Infection

**DOI:** 10.3390/jcm12175542

**Published:** 2023-08-25

**Authors:** Kevin-Arno Koch, David M. Spranz, Fabian Westhauser, Tom Bruckner, Burkhard Lehner, Abtin Alvand, Christian Merle, Tilman Walker

**Affiliations:** 1 Department of Orthopaedic Surgery, University Hospital of Heidelberg, Schlierbacher Landstrasse 200a, 69118 Heidelberg, Germany; kevin-arno.koch@med.uni-heidelberg.de (K.-A.K.); david.spranz@med.uni-heidelberg.de (D.M.S.); fabian.westhauser@med.uni-heidelberg.de (F.W.); burkhard.lehner@med.uni-heidelberg.de (B.L.); 2 Institute of Medical Biometry and Informatics, University of Heidelberg, Im Neuenheimer Feld 305, 69120 Heidelberg, Germany; bruckner@imbi.uni-heidelberg.de; 3 Adult Hip and Knee Service, Nuffield Orthopaedic Centre, Oxford University Hospitals NHS Foundation Trust, Windmill Road, Headington, Oxford OX3 7LD, UK; abtin.alvand@wolfson.ox.ac.uk; 4 Orthopaedic Centre Paulinenhilfe, Diakonie-Klinikum Stuttgart, Rosenbergstraße 38, 70176 Stuttgart, Germany; christian.merle@diak-stuttgart.de

**Keywords:** total knee arthroplasty, revision knee arthroplasty, periprosthetic joint infection, two-stage exchange arthroplasty, reinfection, host status, rotating-hinge implant, mortality

## Abstract

(1) Background: In the treatment of periprosthetic joint infection (PJI), the individual host status and previous surgical procedures appear to have a relevant influence on success rates and clinical outcome of knee revision surgery. Current data about the predictive value are limited in this subgroup of patients. (2) Methods: Retrospectively, 107 patients (109 knees) undergoing two-stage exchange knee arthroplasty for PJI using a rotating-hinge design with at least two years follow-up. The cumulative incidence (CI) for different endpoints was estimated with death as competing risk. Univariate and multivariate analyses for potential predictive factors were performed. Patient-related outcome measures (PROMs) for clinical outcome were evaluated. (3) Results: At 8 years, the CI of any revision was 29.6%, and of any reoperation was 38.9%. Significant predictors for risk of re-revision were the Charlson Comorbidity Index (CCI) and the number of previous surgical procedures prior to explanation of the infected implant. The functional and clinical outcome demonstrated acceptable results in the present cohort with a high comorbidity level. (4) Conclusions: A compromised host status and multiple previous surgical procedures were identified as negative predictors for re-revision knee surgery in the treatment of PJI. Reinfection remained the major reason for re-revision. Overall mortality was high.

## 1. Introduction

Regarding the aging population and steadily increasing numbers of primary total joint procedures, the complexity of cases and number of (re-)revision total knee procedures will substantially rise [1,2]. Estimated projections predict a sevenfold increase from 2005 to 2030 [3]. Periprosthetic joint infection (PJI) is a devastating complication and a major reason for (re-)revision surgery. While infection occurs in 1–2% of cases after primary total knee arthroplasty [4,5,6,7,8], higher infection rates exceeding 10% [9,10] are reported for revision TKA. Revisions for infection carry an even higher risk of subsequent re-revision than revision for aseptic reasons [11]. From the patients’ perspective, PJI is associated with an increased morbidity and mortality [12,13] as well as pain, poor functional outcomes, and reduced quality of life [14,15,16]. In the treatment of PJI, the orthopaedic surgeon must consider both patient and surgical factors and a multi-disciplinary setting is required. The two-stage approach remains the gold standard with the highest success rates [5,17] and variable implant survival rates [18,19,20].

Recent registry data have demonstrated that the early risk for re-revision is substantial within the first two years following revision surgery [21]. It has been shown that the host status of the patient must be considered as a relevant prognostic factor. There are several reports on implant survival and revision rates for various implant types in the treatment PJI following knee arthroplasty [17,22]. However, many available studies lack crucial information about medical comorbidities, the number of previous operations and details of the revision procedures. Several studies do not report on overall reoperation rates, which is crucial from the patients’ perspective. There are very few studies that have demonstrated a predictive value of these factors for patient reported outcome [23,24,25,26].

The purpose of the current study was to report on the clinical and radiological results, as well as mid-term survivorship of two-stage revision knee arthroplasty performed at a tertiary referral centre for periprosthetic joint infection using a rotating-hinge implant design. Furthermore, potential predictive factors for re-revision surgery were explored.

## 2. Patients and Methods

### 2.1. Study Cohort

In the present retrospective cohort study, we reviewed all patients who had undergone two-stage revision TKA for PJI at our institution between January 2001 and December 2015 using a rotating-hinge TKA at time of re-implantation. The institutional review board approved all procedures (S-152/2017) and the study was conducted in accordance with the Helsinki Declaration of 1975, as revised in 2013 [27]. Informed consent was obtained from all participating patients prior to inclusion in the study.

**Figure 1:**
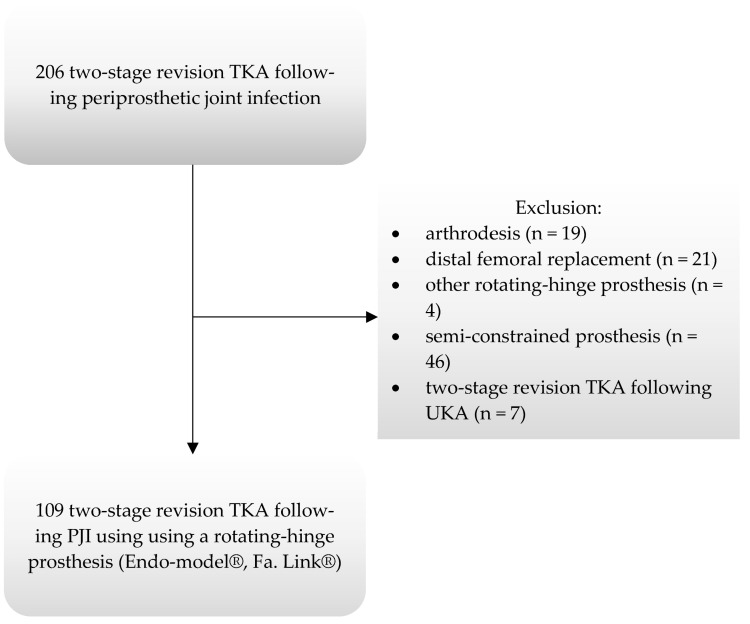
Study cohort.

Patients were prospectively monitored following the index surgery with the institutions’ joint replacement registry, but the study design was set up retrospectively. Exclusion criteria were defined as cases involving the use of a distal femoral and/or proximal tibial replacement, oncologic indications for primary TKA, or patients who had received therapy with debridement and implant retention (DAIR) or reimplantation using non- or semi-constrained implants. For the diagnosis of PJI the patients’ medical history, physical examination, laboratory and radiographic findings, and joint fluid aspiration results were evaluated [28]. Of 206 consecutive patients revised for periprosthetic joint infection in the study period, 107 patients (109 knees) fulfilled the inclusion criteria (Figure 1). Patient demographics, medical comorbidities, patient- and surgery-related factors, and implant details were collected and are presented in Table 1 and Table 2.

### 2.2. Revision Protocol

All procedures were carried out using a two-stage protocol. First-stage treatment included implant and cement removal, radical debridement of infected and necrotic soft tissue and bone, and insertion of an antibiotic polymethylmethacrylate (PMMA) spacer. Intraoperative fluid and tissue samples were collected in all cases and sent for microbiological (+/− histological) analysis. After a period of 6 weeks of antibiotic therapy and an antibiotic-free interval of 2 weeks, a clinical reassessment was performed including a joint aspiration and a laboratory control. In patients with a positive culture or clinical/laboratory signs of a persisting infection, a re-debridement with a spacer exchange was performed (22 cases; 21%). In all other patients, the re-implantation was planned directly after the confirmation of a negative aspiration. Standardized preoperative planning of the prosthesis size and position was performed in all patients on calibrated radiographs. At time of re-implantation, a fully cemented rotating-hinge implant system was used. At our institution, the Endo-model^®^ rotating-hinge implant system (Link^®^, Waldemar Link GMBH and Co., Hamburg, Germany) was used, which is characterized by a metal hinge, which allows flexion-extension and axial rotations and thus reduces the forces acting on the prosthesis anchorage. Cases performed after 2011 utilised the modular version (Endo-Model^®^ SL^®^, Link^®^, Waldemar Link GMBH and Co., Hamburg, Germany) of the implant, which allowed for individual determination of stem length. The majority of patients (90%) received the complete two-stage exchange procedure at our institution according to the revision protocol for PJI, whereas 10% had the first stage explantation at an external institution. The reconstruction was performed using fully cemented fixation of diaphyseal stems and meta-epiphyseal components of the implant. In 55 cases (51%), tibial augments were used. In 5 procedures, metaphyseal cones were utilized for femoral reconstruction. Patella resurfacing was performed in 19 patients (17%).

Antibiotic treatment for both first-stage treatment and second-stage treatment was administered for 6 weeks postoperative. According to sensitivity testing, penicillins and cephalosporines for intravenous treatment and penicillins, lincomycins, and fluoroquinolones for oral treatment were used. For multi-resistant bacteria, glycopeptides, carbapenems, and oxazolidonines were administered.

### 2.3. Clinical Follow-Up and Radiographic Assessment

Clinical and radiographic follow-up examinations were recommended in regular intervals at 6 weeks, 12 weeks, 1 year, 3 years, 5 years, and then every 5 years thereafter. At the most recent follow-up, the following patient related outcome measures (PROMs) were assessed: American Knee Society Score (AKSS) [29], Oxford Knee Score (OKS) [30], and Visual analogue scale (VAS) [31]. Patients who were unable to attend clinical follow-up were contacted via telephone or via mail to complete the questionnaires. In deceased patients, information on further revision procedures or complications between the last clinical follow-up and death was obtained using information from their general practitioner and hospital records.

Radiographs were examined by two independent experienced orthopaedic surgeons (C.M., D.S.), blinded to outcome. The first postoperative and subsequent serial anteroposterior and lateral radiographs of the knee were examined to determine subsidence, radiolucent lines, osteolysis, heterotopic ossifications, and signs of loosening. The threshold for subsidence was defined as >2 mm on serial radiographs. Radiolucent lines were defined as progressive lines of >1 mm adjacent to the implant-cement or cement-bone interface. Osteolysis was defined as progressive areas of bone resorption or endosteal erosion. Implant loosening was diagnosed if radiolucent lines of over 2 mm were present around the entire implant or if implant migration by >2 mm or a change in implant axis >3° was observed.

### 2.4. Statistical Analysis

Following exploratory data analysis, implant survival for the endpoints revision (defined as removal or exchange of at least one of the components), reinfection, aseptic loosening, and reoperation was estimated, calculating the cumulative incidence of revision with 95% confidence intervals (CI) with death as competing risk. Survival was calculated up to 8 years, with a minimum of 23 knees still being at risk. Univariate analysis using a Cox proportional hazards regression model was performed to screen predictive risk factors for revision. Subsequently, a Cox proportional hazards regression model was used for multivariate analysis, including age at operation, BMI, Charlson Comorbidity Index (CCI), and number of previous operations before explantation as continuous independent variables. We considered *p*-values of <0.05 to be statistically significant. SPSS Version 24.0 (IBM SPSS Statistics, IBM, Armonk, NY, USA) and GraphPad Prism Version 6.0 (GraphPad Software, San Diego, CA, USA) were used to record and analyse the collected data.

## 3. Results

**Figure 2:**
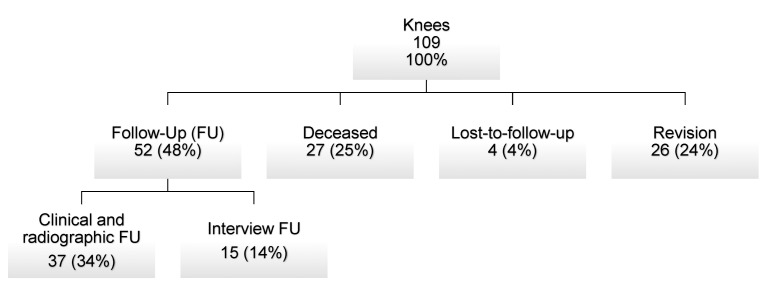
Distribution of knees at most recent follow-up.

A total of 109 two-stage exchange arthroplasties for periprosthetic joint infection using a rotating-hinge-knee in 107 patients were included. Mean follow-up was 58 months (range: 0–188 months); patient demographics as well surgical and implant-related factors are summarized in Table 1. At the most recent follow-up, 52 of 109 knees (48%) were available for clinical assessment. Of those, 37 patients underwent clinical and radiological examination, whereas 15 patients had a standardized phone interview at final follow-up. A total of 27 patients (25%) died without need for further revision surgery. Four patients were lost to follow-up (3%) (Figure 2).

### 3.1. Survival Analysis

**Figure 3:**
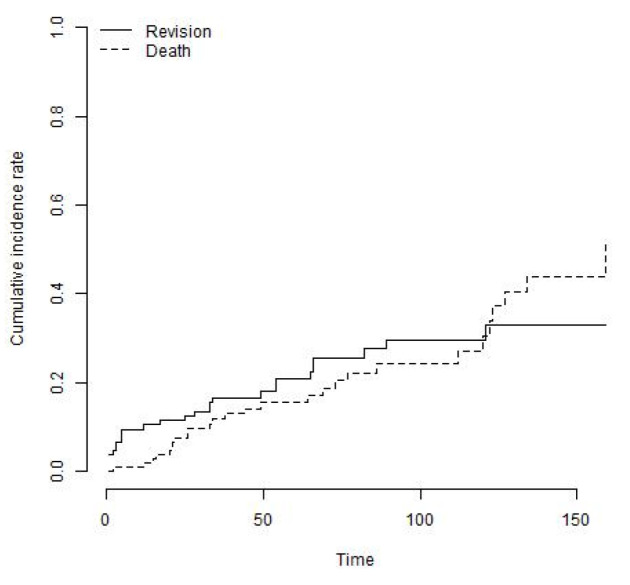
The cumulative incidence of the endpoint revision for any reason with death as a competing risk was 29.6% (95% CI: 19.3% to 39.9%) at 8 years.

**Figure 4:**
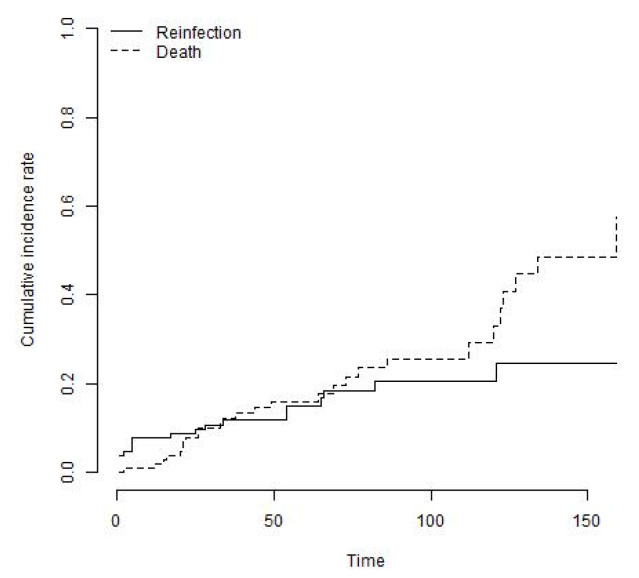
The cumulative incidence of the endpoint revision for recurrent infection with death as a competing risk was 20.6% (95% CI: 11.3% to 29.9%) at 8 years.

**Figure 5:**
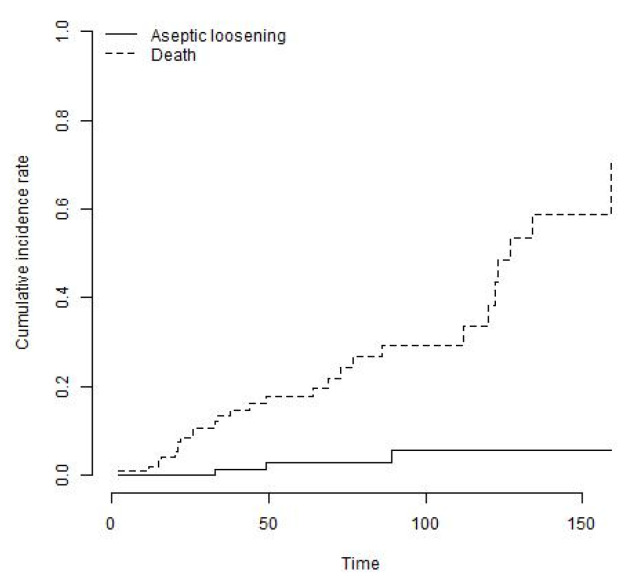
The cumulative incidence of the endpoint revision for aseptic loosening with death as a competing risk was 5.6% (95% CI: 0% to 12.3%) at 8 years.

26 knees (24%) underwent a re-revision procedure. In three patients (11.5%), revision was performed for aseptic loosening, two of those for aseptic loosening of the femoral component and one for the tibial component. One revision (3.8%) was performed for mechanical failure of the locking mechanism and another one for a periprosthetic fracture of the femur. Three revisions (11.5%) were performed at external institutions for unknown reasons. Eighteen knees (69.2%) were revised for recurrent deep infection (Table 3). The mean time to reinfection after reimplantation was 33 months (range, 1 to 121 months) and 42% of re-infections occurred within the first year following index surgery. The cumulative incidence of the endpoint revision for any reason with death as a competing risk was 29.6% (95% CI: 19.3% to 39.9%) at 8 years (Figure 3). The cumulative incidence of the endpoint revision for recurrent infection was 20.6% (95% CI: 11.3% to 29.9%) at 8 years (Figure 4). The cumulative incidence of the endpoint revision for aseptic loosening was 5.6% (95% CI: 0% to 12.3%) at 8 years (Figure 5).

**Figure 6:**
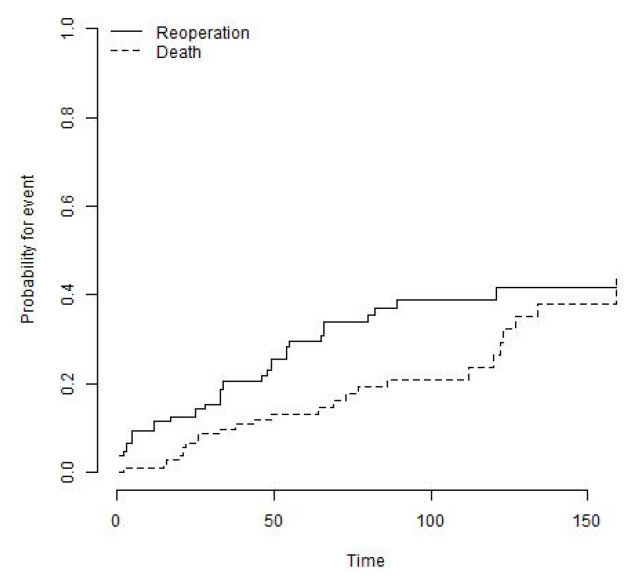
The cumulative incidence of the endpoint all reoperations was 38.9% (95% CI: 28.2% to 49.6%) at 8 years.

Additionally, nine re-operations (8.2%) with implant retention were performed for open reduction and internal fixation (ORIF) (three knees), wound healing issues/postoperative haematoma (four knees), and patellar maltracking (two knees) (Table 3). The cumulative incidence of the endpoint all reoperations was 38.9% (95% CI: 28.2% to 49.6%) at 8 years (Figure 6).

### 3.2. Mortality

**Figure 7:**
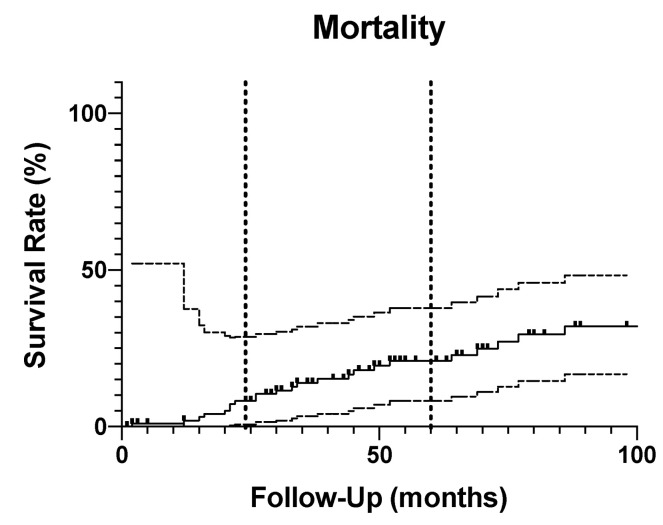
The mortality rate estimated with the Kaplan–Meier analysis was 9.2% (95% CI: 4.2% to 15.9%) at 2 years and 21.0% (95% CI: 13.9% to 31.7%) at 5 years.

In total, 33 patients (30.2%) were deceased at the most recent follow-up. Of those, 27 patients died without need for further re-revision surgery, whereas six patients underwent prior re-revision. The mortality rate estimated with the Kaplan–Meier analysis was 9.2% (95% CI: 4.2% to 15.9%) at 2 years and 21.0% (95% CI: 13.9% to 31.7%) at 5 years (Figure 7).

### 3.3. Regression Analysis

Multivariate analysis identified CCI (HR: 1.200 [95% CI: 1.001–1.437]; *p* = 0.049) and number of previous operations before explantation (HR: 1.146 [95% CI: 1.012–1.298]; *p* = 0.032) as significant predictive factors for re-revision (Table 4). Sex, age, BMI, preoperative positive cultures, use of modular implants, use of augments or patella resurfacing, perioperative bloodloss, operative time, and ASA score did not show any significant influence on the risk for re-revision surgery in the present cohort.

### 3.4. Microorganisms Causing Reinfection

In 77% of patients, a pathogen organism could be isolated at the time of index surgery. In cases with recurrent infection (n = 18), a positive culture of a pathogen microorganism could be isolated in 13 cases (72%). In 5 cases (28%), decisive clinical and radiological signs of PJI led to revisions surgery. In 5 of 13 patients (38%) with positive cultures, the same pathogen leading to reinfection was detected and classified as a persistent infection. Microorganisms for persistent infection were *Enterococcus faecalis* (two knees), *Staphylococcus aureus* (one knee), *Staphylococcus caprae* (one knee), and *Candida parapsilosis* (one knee).

### 3.5. Clinical Outcome

At the most recent follow-up, the mean postoperative knee flexion was 93° (SD: 17°, range: 40° to 130°). The mean Knee Society score was 71 points (SD: 21 points, range: 26 to 97 points); 43% of the patients showed excellent outcome (AKSS > 80) and 37% showed poor outcome (AKSS < 60). The mean Knee society functional score was 38 points (SD: 27 points, range: 0 to 100 points). Clinical evaluation according to the OKS demonstrated very good results in 12% of the patients (48–40 points), good results in 23% (39–30 points), fair in 34% (29–20 points), and poor in 31% (less than 20 points), showing a mean OKS of 26 points (SD: 10 points, range: 8 to 47 points). Patients’ self-assessment based on the VAS (0–10) for pain revealed a median score of 4 (range 0 to 10).

### 3.6. Radiographic Findings

In total, 37 patients were available for radiological assessment. Of those, three knees (8%) showed radiographic evidence of implant loosening, one for the femoral component and two for the entire implant. At the most recent follow-up, these impending revisions were not revised due to limited overall health condition without medical clearance for surgery. Of the remaining patients, progressive radiolucent lines were detected in 21 cases (62%). For the femoral component, epiphyseal radiolucent lines were observed in one patient, epimetadiaphyseal radiolucent lines in three patients, and epimetadiaphyseal radiolucent lines in one patient. For the tibial component, epiphyseal radiolucent lines were observed in one patient, epimetadiaphyseal radiolucent lines in four patients, and epimetadiaphyseal radiolucent lines in two patients. For both components, epimetaphyseal radiolucent lines were observed in six patients and epimetadiaphyseal radiolucent lines in three patients. Overall, none of those patients showed clinical signs of loosening or instability.

Heterotopic ossification was seen in three patients, leading to a functional ankylosis in one patient. Radiographically, two patients showed patellar maltracking. No signs of osteolysis were detected.

## 4. Discussion

In the present study, we evaluated the clinical and radiological mid-term results of two-stage exchange arthroplasty for periprosthetic joint infection using a cemented rotating-hinge TKA (Endo-model^®^, Fa. Link^®^) in a retrospective cohort of patients with a high level of comorbidity as well as multiple previous revisions. The cumulative incidence for the endpoint “any revision surgery” was 29.6% at 8 years (95% CI: 19.3% to 39.9%) and for the endpoint “any reoperation” the cumulative incidence was 38.9% at 8 years (95% CI: 28.2% to 49.6%). The main cause for re-revision surgery was recurrent periprosthetic joint infection with a cumulative incidence for the endpoint “reinfection” of 20.6% at 8 years (95% CI: 11.3% to 29.9%). In comparison, the revision rate for aseptic failure with 5.6% at 8 years (95% CI: 0% to 12.3%) was significantly lower. Using regression analysis, the study identified both the number of previous operations as well as the comorbidity level (as defined by the Charlson Comorbidity Index) as significant predictive factors regarding the risk for further re-revision surgery. Implant revision rates were comparable to previous reports. However, it is also important to consider the most relevant endpoint from the patients’ perspective, i.e., all reoperations, that accounted for approximately 10% additional surgical re-interventions. It is also worth noting that one third of patients had a poor functional outcome. The present data is hence helpful to counsel patients prior to revision for PJI about the probability of future surgeries and the substantial risk of limited joint function.

The eradication of PJI is challenging and requires a multidisciplinary treatment setting, including orthopaedic surgeons, microbiologists, and internal medicine specialists. Regarding the surgical procedure, two-stage exchange arthroplasty is still considered the gold standard in the treatment of PJI [5,17]. Nevertheless, neglecting studies with low patient numbers and/or short-term follow-up, the reinfection rates of previous studies range between 7% and 36% [19,20,23,24,32,33,34,35]. The available literature is difficult to compare because of variable outcome parameters and a substantial variation in definition of success and failure in heterogeneous study cohorts. According to our findings, previous operations and previous revision surgery both showed a statistically significant and clinically highly relevant influence on the re-revision rate. Our results show that every previous operation before explantation increases the risk for re-revision by 15%. Sabry et al. demonstrated a high number of 105 patients (33.4%) with failure to eradicate PJI in their study of 314 patients following two-stage exchange arthroplasty for PJI [24]. In their study, patients with recurrent infection showed an increased number of previous surgeries [24]. Similarly, Pelt et al. found that patients with more than four surgeries between primary TKA and explantation of the prosthesis due to treatment of PJI appear to have a higher risk of failure [32]. Furthermore, in both studies the need for plastic coverage procedures showed a greater risk for re-revision [24,32]. In contrast to these results, Haleem et al. could demonstrate a relatively high 10-year-survivorship of 85% for the endpoint “reinfection” [19]. As 91% of the implants used in their study were posterior-stabilized prosthesis, it can be assumed that these patients had less ligamentary and/or bony defects due to fewer prior operations [19].

Moreover, with an increase of previous revisions, the success rate for PJI is decreasing [36]. While patients without prior revision surgery undergoing treatment for PJI showed a success rate of 89%, patients with at least one revision surgery in prehistory showed a lower success rate of 73% [36]. Taking previous septic revision into consideration, the risk of re-revision rises even more. Mortazavi et al. demonstrated an overall survival rate for re-revision of 72.9% at 90 months in a study of 475 patients (499 knees) following revision surgery [10]. When only patients following two-stage exchange arthroplasty for septic modes were considered, the survival rate dropped to 52.4% at 90 months [23]. These observations demonstrate that previous revision surgery leading to bony and soft tissue deficiencies are associated with higher failure rates and risk for re-revision after treatment of PJI.

Another important factor for a successful treatment of PJI is the patients’ general health status. Concerning risk factors for revision surgery following primary TKA, there are some studies demonstrating that sociodemographic factors such as male sex, smoking, or an elevated BMI have been described as risk factors for revision surgery, while age was not associated with a higher risk for revision [4,37,38,39,40,41]. Furthermore, the influence of comorbidities was examined. While a history of diabetes or rheumatoid arthritis could be confirmed as risk factors for revision surgery [8,39,42,43], high blood pressure or cardiovascular diseases showed no increased impact on the rate of revision surgery [44,45]. Although these factors were mainly described in cohorts of patients following primary TKA, some studies could identify positive tendencies for these factors as predictors for re-revision surgery. Watts et al. were able to demonstrate that patients with a BMI > 40 kg/m^2^ had a 4.45-fold and 4.86-fold risk for subsequent revision and reinfection, respectively, compared with a matched, non-obese cohort [46]. In general, the data for comorbidities for recurrent revision after PJI treatment is very limited. Even though particular comorbidities are associated with a higher severity for PJI, from our perspective the influence is multifactorial. The present study demonstrates that an increase in CCI by one point leads to an increased risk for re-revision by 15% and an increased risk for reinfection by 28%. Similarly, Cochran et al. have demonstrated that a higher CCI is associated with a higher risk for reinfection in patients receiving a primary TKA [5]. In their study, the adjusted risk of reinfection was 48% higher in patients with a CCI of 5 and more, compared to patients with a CCI of 0 [5]. Furthermore, Mortazavi et al. showed that patients with a CCI > 3 were 2.48 times more likely to develop reinfection following revision TKA for any reason compared to patients with a CCI of 0 to 2 [10].

The distribution of isolated pathogens in our study, with a high number of staphylococceal infections, predominantly *S. epidermidis*, compares well to previous data in the literature [23,26,47,48,49]. In 23% of our cases, no pathogen could be isolated; this is attributed to antibiotic use prior to sample taking. Similar to Malekzadeh et al. and Kim et al. [50,51], there were no differences in the outcome regarding re-revision (*p* = 0.952) or reinfection (*p* = 0.744) between patients with positive or negative cultures. In a large study cohort by Zmistowski et al., it was demonstrated that recurrent PJI were mainly caused by a different pathogen, whereas in only 29 of 92 patients (31.5%) the same pathogen could be identified, which was classified as a persistent PJI [52]. Similar to these results, our results demonstrate a persistence of PJI in 38%. Therefore, we assume PJI failure is predominately related to a secondary infection instead of failed surgical treatment. This may be related to a generally poor medical condition concerning soft tissue and comorbidities.

The functional outcome after revision TKA is associated with inferior results when compared to primary TKA [53,54,55] due to the presence of bony defects and poor soft tissue [56]. Studies report similar functional results between aseptic and septic study cohorts if local tissue damage is comparable [57,58,59,60]. Compared to existing data, the functional and clinical scores in our study demonstrate similar results except for the KSFS [57,60,61]. This could be explained by the substantial comorbidities and high number of previous surgeries leading to impaired soft tissue condition in our study group. Furthermore, the use of a constraint prosthesis must be interpreted as another relevant factor for functional outcome. While Konrads et al. showed a mean OKS of 33.9 in 52 patients after PJI [59], Bejon et al. and Matar et al. demonstrated lower mean scores of 27 and 26, respectively, in patients with a compromised host status [36,62]. These results match the mean OKS of 26 in the present study.

As a tertiary referral centre, the majority of our patients presented numerous medical comorbidities as well as several prior surgical procedures, leading to a poor local bone/tissue stock. Moreover, in the current study, only patients with rotating-hinge implants were used to limit potential bias as a result of different implant designs. In this context, the present results, with a cumulative incidence for the endpoint “any revision surgery” of 29.6% at 8 years (95% CI: 19.3% to 39.9%) and for the endpoint “reinfection” of 20.6% at 8 years (95% CI: 11.3% to 29.9%), are on an acceptable level when compared to previously reported numbers.

The study has several limitations, including the retrospective study design as well as a missing concluded guideline for the surgical and medical treatment for infection. Highly variable patient-related factors, prior treatments, and surgical procedures must be considered as confounders. Additionally, the decision of the treatment strategy was made by different orthopedic surgeons with different experiences in knee arthroplasty revision surgery and a potential preoperative selection.

## 5. Conclusions

In conclusion, two-stage exchange arthroplasty for periprosthetic joint infection using a cemented rotating-hinge TKA outlined acceptable mid-term results regarding clinical function as well as survival rates with the endpoint “re-revision surgery” and “reinfection”. Recurrent periprosthetic joint infection was the major reason for subsequent revision surgery whereas aseptic loosening appeared of minor importance in the chosen competing risk analysis. A high number of previous operations as well as a high Charlson Comorbidity Index were clinically relevant predictive factors regarding the risk for further revision surgery. Mortality at 5 years was high in the present cohort. Patients with multiple comorbidities and a high number of previous operations need to be informed about the increased risks of PJI treatment with regard to re-revision risk, suboptimal in functional outcome, and mortality risk.

## Figures and Tables

**Table 1:** Patient demographics, medical comorbidities, patient- and surgery-related factors, and implant details.

Demographics	
Number of knees	109 (100%)
Age (mean, SD), in years	69.61 (9.35)
Gender n (%)	
Male	55 (50.5%)
Female	54 (49.5%)
Body mass index (mean, SD)	30.96 (5.62)
Comorbidity Scores	
ASA score (%)	
ASA I	2 (1.8%)
ASA II	27 (24.8%)
ASA III	75 (68.8%)
ASA IV	5 (4.6%)
Charlson Comorbidity Index	
0	20 (18.3%)
1–2	36 (33.0%)
3–4	26 (23.9%)
5	27 (24.8%)
Surgical-related factors	
Two-stage revision	
Complete n (%)	98 (90%)
Reimplantation n (%)	11 (10%)
Pathogen isolation at index surgery	
Gram-positive	70 (64%)
Staphylococci	51 (47%)
*Staphylococcus aureus*	14 (13%)
Methicillin-resistant *Staphylococcus aureus*	1 (1%)
Coagulase-negative staphylococci/*Staphylococcus epidermidis*	27 (25%)
Methicillin-resistant *Staphylococcus epidermidis*	2 (2%)
Streptococci	6 (6%)
Enterococci	4 (4%)
Other gram-positive	9 (8%)
Gram-negative	8 (7%)
Fungus	2 (2%)
Poly-microbial	4 (4%)
No organism growth	25 (23%)
Blood loss (mean, SD), (ml)	1339 (915)
Operating time (mean, SD), (minutes)	188 (58)
Previous operations (mean, SD)	4.94 (2.92)
Previous revision procedure	
Yes	62 (56.9%)
Aseptic	23 (21.1%)
Septic	39 (35.8%)
No	47 (43.1%)
Surgical procedures between explantation and reimplantation	
Yes	22 (20.2%)
No	87 (79.8%)
Implant-related factors	
Generation of RHK	
Modular n (%)	55 (50.5%)
Non-modular n (%)	54 (49.5%)
Retropatellar replacement	
With n (%)	19 (17.4%)
Without n (%)	90 (82.6%)
Tibial/Femoral Augments	
With n (%)	55 (50.5%)
Without n (%)	54 (49.5%)

**Table 2:** Comorbidities in study cohort according to Charlson Comorbidity Index.

Comorbidity	Total n = 109 (100%)
Myocardial infarction	13 (12%)
Congestive heart failure	34 (31%)
Peripheral vascular disease	5 (5%)
Cerebrovascular accident or transient ischemic attacks	12 (11%)
Dementia	0 (0%)
Chronic obstructive disease	26 (24%)
Connective tissue disease	11 (10%)
Peptic ulcer disease	55 (50%)
Liver disease	7 (6%)
mild	6 (5%)
moderate to severe	1 (1%)
Diabetes mellitus	34 (31%)
uncomplicated	26 (24%)
End-organ damage	8 (7%)
Hemiplegia	3 (3%)
Moderate to severe chronic kidney disease	40 (37%)
Solid tumour	4 (4%)
Leukemia	0 (0%)
Lymphoma	0 (0%)
Metastasic tumour	1 (1%)
AIDS	0 (0%)

**Table 3:** Causes of re-revisions and reoperations.

	Number of Knees
**Re-Revisions**	26
Recurrent deep infection	18
Aseptic loosening	3
Femoral component	2
Tibial component	1
Implant failure	1
Periprosthetic fracture	1
Revision for unknown reason	3
**Reoperations (excluding reasons leading to revision)**	9
Open reduction and internal fixation (ORIF)	3
Wound healing/postoperative haematoma	4
Patellar maltracking	2

**Table 4:** Multivariate Cox regression model for the likelihood of any revision dependent on specific risk factors.

Risk Factor	Hazard Ratio (95% CI)	*p*-Value
Age at operation	1.010 (0.953–1.070)	0.733
BMI	0.993 (0.917–1.076)	0.869
Charlson Comorbidity Index	1.200 (1.001–1.437)	0.049
Previous operations	1.146 (1.012–1.298)	0.032

## Data Availability

The datasets used and analyzed during the current study are available from the corresponding author on reasonable request.
